# Measuring the Destination Accessibility of Cycling Transfer Trips in Metro Station Areas: A Big Data Approach

**DOI:** 10.3390/ijerph16152641

**Published:** 2019-07-24

**Authors:** Xueying Wu, Yi Lu, Yaoyu Lin, Yiyang Yang

**Affiliations:** 1 School of Architecture, Harbin Institute of Technology, Shenzhen 518000, China; 2 Department of Architecture and Civil Engineering, City University of Hong Kong, Kowloon Tong, Hong Kong, China; 3 City University of Hong Kong Shenzhen Research Institute, Shenzhen 518057, China

**Keywords:** bicycle, destination accessibility, distance decay, bicycle-metro integration, bicycle-sharing

## Abstract

Cycling is a green, sustainable, and healthy choice for transportation that has been widely advocated worldwide in recent years. It can also encourage the use of public transit by solving the “last-mile” issue, because transit passengers can cycle to and from transit stations to achieve a combination of speed and flexibility. Cycling as a transfer mode has been shown to be affected by various built environment characteristics, such as the urban density, land-use mix, and destination accessibility, that is, the ease with which cyclists can reach their destinations. However, cycling destination accessibility is loosely defined in the literature and the methods of assessing cycling accessibility is often assumed to be equivalent to walking accessibility using the same decay curves, such as the negative exponential function, which ignores the competitive relationship between cycling and walking within a short distance range around transit stations. In this study, we aim to fill the above gap by measuring the cycling destination accessibility of metro station areas using data from more than three million bicycle-metro transfer trips from a dockless bicycle-sharing program in Shenzhen, China. We found that the frequency of bicycle-metro trips has a positive association with a trip distance of 500 m or less and a negative association with a trip distance beyond 500 m. A new cycling accessibility metric with a lognormal distribution decay curve was developed by considering the distance decay characteristics and cycling’s competition with walking. The new accessibility model outperformed the traditional model with an exponential decay function, or that without a distance decay function, in predicting the frequency of bicycle-metro trips. Hence, to promote bicycle-metro integration, urban planners and government agencies should carefully consider the destination accessibility of metro station areas.

## 1. Introduction

As an environmentally friendly transportation mode, cycling can reduce traffic congestion and air pollution in urban areas [1,2]. It also attracts considerable research attention because of its low cost, great flexibility, and moderate distance range [2,3]. It is feasible to incorporate cycling, especially for transportation purposes, into urban residents’ daily routine. Half of global private vehicle trips shorter than five km could be replaced by cycling trips of up to 15 min [4]. In addition, as an active transportation mode, cycling can help cyclists achieve the recommended level of physical activity and maintain their health; various health benefits associated with regular cycling have been identified, including reducing body weight and the risk of obesity and type II diabetes and improving mental health [5,6].

Cycling can also promote the use of public transit, a concept often called bicycle-transit integration. The trip distance between one’s home or office and a transit station is a major barrier to the promotion of transit ridership in large cities [7,8]; a longer distance may encourage the use of a private vehicle rather than transit [9]. Bicycle–transit integration can solve the “last-mile” issue, because cycling can cover larger areas than walking to efficiently feed transit passengers to and from metro stations [9,10,11,12]. To promote both cycling and transit use, bicycle–transit integration has already been advocated by many governments in both developed countries such as the United States, Australia, the Netherlands, and Denmark and developing countries such as Brazil, Colombia, and China [13].

Understanding the effects of the built environment characteristics on cycling behaviors can help policymakers to alter the built environment to promote bicycle–transit integration [14,15,16]. The cycling destination accessibility of transit stations, which is defined as the degree to which a person can cycle to her or his destination from a transit station or vice versa, is believed to be the primary determinant of transit use [17]. Only in the presence of such accessibility will a user consider other factors such as cost, comfort, or security [18]. However, cycling accessibility has received less attention than walking accessibility. Some researchers tend to regard cycling accessibility and walking accessibility as the same concept, often termed “active accessibility” or “non-motorized accessibility” [19,20], so the subtle differences between the two concepts are often ignored.

Gravity-based measures are often used to assess both walking accessibility and cycling accessibility due to their relative ease of calculation and interpretation [19,20,21]. Several cost decay functions have been used as the impedance function in evaluating accessibility, such as threshold functions (e.g., step function) and smooth cost decay functions (e.g., negative exponential, Box–Cox, or Tanner functions) [22]. However, distance decay functions have been adopted from walking accessibility, and their feasibility has yet to be rigorously tested.

In addition, studies have often relied on questionnaires or travel diaries to collect cycling trip data; those methods have some inherent limitations, such as high cost, small sample size and study area, and data inaccuracy due to recall bias. The emerging bicycle-sharing program equipped with global position system (GPS) devices provides a big data source and research opportunity to scrutinize cycling behaviors.

To address these research gaps, we assess cycling destination accessibility around metro stations using cycling trip data from a large-scale bicycle-sharing program in Shenzhen, China. The distance impedance is examined, and a corresponding decay function is proposed. The proposed accessibility model is further compared with existing models in terms of the performance of modeling real cycling usage. The relationships between the built environment and cycling trips to and from metro stations are also discussed.

## 2. Literature Review

### 2.1. Built Environment and Cycling Behavior

Built environment characteristics have been recognized as significant determinants of cycling trips to and from metro stations; these characteristics include the presence of cycling infrastructure, urban density, land-use mix, street connectivity, destination accessibility, and aesthetics [9,23,24,25].

Urban density and land-use mix are both important indicators of cycling behaviors. It has been shown that population density and destination density (e.g., job density, retail density, and business density) have positive relationships with public bicycle use [26,27,28,29]. More facilities mean more chances for people to choose to cycle. Similarly, the land-use mix is also important to encourage cycling [30,31]. For example, the presence of many daily living facilities (e.g., convenience stores, fast food restaurants, and hospitals) in a residential area could increase cycling rates [32].

The presence of cycling infrastructure directly affects cycling behavior. Generally, the presence of bicycle lanes contributes to a higher likelihood of cycling [32]. In particular, cyclists with low incomes and those older than 30 years seem to be more sensitive to bicycle network connectivity [33]. Moreover, different types of bicycle trails, lanes, and paths may have different effects on cycling behavior [34]. On-road bicycle lanes with bicycle parking are more attractive to cyclists than off-road bicycle trails [35]. It should be noted that some researchers have found that the supply of bicycle lanes has a limited effect or even no effect on the cycling rate [36].

Aesthetic factors are essential in encouraging cycling behavior, in addition to factors related to urban density, the land-use mix, and the cycling infrastructure. Among the aesthetic factors, the presence of urban greenness plays an important role in promoting cycling, because the presence of urban greenness often gives cyclists a more pleasant experience [1,37,38,39].

Cycling destination accessibility reflects a person’s ability to reach destinations via cycling. It is indirectly or directly covered in some built environment measures. For example, density and land-use mix can be regarded as proxies for overall accessibly, because denser areas with mixed land use may provide more cycling destinations within cycling distance. The distance to the closest facilities is an explicit measure of accessibility. For transit riders who use cycling as a transfer mode, the distance from their home to the transit station is an important factor [40,41]. The maximum cycling distance between one’s home and a transit station ranges from 1.2 to 3.7 km [42,43].

### 2.2. Gravity-Based Cycling Accessibility

In addition to cycling accessibility based on a fixed distance threshold, studies often measure dynamic accessibility on the basis of certain distance-decay functions [20]. Cycling demand is assumed to be a compromise between the benefit gained from destination/activity opportunities and the cost to reach them from a given origin. The formula is expressed as Equation (1).

(1) $$A_{i}=\sum a_{j}f\left(t_{i,j}\right)$$

where *A_i_* represents the accessibility to place *i*; *a_j_* denotes the activity in place *j*; *t_i,j_* represents the travel impedance between place *i* and *j*, which can be considered as time, distance, or other cost; and *f*(*t_i,j_*) is an impedance function that measures the spatial separation between *i* and *j*.

It should be noted that the key component of this formula is the definition of the impedance between two locations. An array of distance-decay functions have been used in empirical studies. Prins, et al. [44] applied an exponential distance-decay function to estimate shopping trips of older adults, based on GPS data from the walking and cycling trips they generate. Iacono, Krizek and El-Geneidy [19] chose a negative exponential distance-decay function based on the frequency distribution of trip lengths among workplaces, shopping centers, schools, recreation facilities, and restaurants.

Walking accessibility and cycling accessibility are usually not distinguished from each other, and the combination of the two is sometimes referred to as “active accessibility” or “non-motorized accessibility”. Although walking and cycling share some common features, they also have marked dissimilarities [45,46]. For example, both are human-powered modes that require a certain physical capability. However, the travel speed and distance range of cycling exceed those of walking. Walking, in turn, is more flexible than cycling and requires no special equipment. Previous studies have often neglected the fact that walking and cycling opportunities may compete with each other when both are available for short trips. Hence, people can choose one transport mode over the other according to their willingness and cost. Therefore, we argue that distance-decay functions for cycling and walking should be estimated separately.

### 2.3. Public Bicycle-Sharing Systems

Public bicycle-sharing systems have often been advocated to encourage both cycling and transit use because transit passengers can cycle to and from the transit stations [47,48]. The bicycle-sharing system, especially with free-floating bicycles, has recently expanded dramatically in China and other countries. For example, the total number of free-floating bicycles in China surpassed 4 million by March 2017 [49].

Free-floating public bicycles allow users to access bicycles at nearly any location and avoid the necessity of docking stations and kiosk machines used for docked bicycles [50]. More importantly, cyclists’ behavior (e.g., the starting and ending location, route choice, and trip distance) can be accurately recorded with built-in GPS devices. Given the large number of users of free-floating public bicycles, these detailed cycling behavior data provide researchers with an attractive alternative to explore the relationship between cycling and the built environment.

Previous studies have used cycling data from free-floating bicycle-sharing systems for the following topics: Demand forecasting and bicycle redistribution issue, users’ travel patterns, and determinants of the use of free-floating public bicycles. For example, Pal, Zhang and Kwon [50] analyzed the mobility patterns and imbalance of free-floating bicycle-sharing systems by analyzing historical trip and weather data to help the system operator to make better-informed decisions. Leonardo, et al. [51] forecasted the trend for bicycle use in every zone of London and consequently enhanced the relocation procedure by generating spatiotemporal clusters of the usage patterns of the available bicycles. Shen, Zhang and Zhao [25] adopted spatial autoregressive models to analyze the spatiotemporal patterns of bicycle use and explored the impact of the fleet size, the surrounding built environment, access to public transportation, the bicycle infrastructure, and the weather conditions. Li, et al. [52] explored the patterns of cycling behavior and found that free-floating bicycles were mainly used for short trips, especially for short commuting trips to work or school. 

Although the main function of free-floating public bicycles is to integrate with public transit stations, especially metro stations, to solve the “last-mile” issue, few studies have examined cycling transfer trips in metro station areas. Liu and Lin [53] addressed the influence of free-floating public bicycles on the catchment areas of metro stations and explored the factors associated with the sizes of bicycle catchment areas. To our knowledge, few studies have assessed cycling accessibility with cycling data from a free-floating bicycle-sharing system.

### 2.4. Research Gaps and Our Research Objectives

In sum, two research gaps exist in cycling accessibility assessment. First, few studies have distinguished cycling accessibility and walking accessibility. The methods of assessing cycling accessibility were mainly adopted from methods of assessing walking accessibility. Walking and cycling are both important modes of active transportation. However, cycling has a higher financial cost and a different time cost (i.e., search for and unlock a bicycle) relative to walking, especially for short trips. People prefer walking to cycling when the trip is short. Hence, competition between cycling and walking exists within a certain distance threshold. The willingness to use cycling does not always decrease as the distance increases. It is thus crucial to assess cycling accessibility.

Second, most studies have relied on survey data to assess cycling behavior, which is inefficient and includes a limited number of participants and study areas [54]. Public bicycle-sharing systems can collect enormous amounts of data on cycling behaviors from a vast number of participants. This new data source gives researchers a chance to measure cycling accessibility on a large geographic scale.

In this study, we aim to fill these gaps by measuring the cycling destination accessibility of metro station areas in Shenzhen, China. First, the specific distance-decay function was estimated with cycling data from a dockless bicycle-sharing system. The performance of the proposed distance-decay function was then compared with the existing distance-decay function in terms of fitting with the number of cycling trips to and from metro stations. We also discuss the potential implications of our results for the transportation planning process.

## 3. Materials and Methods 

### 3.1. Study Area

**Figure 1:**
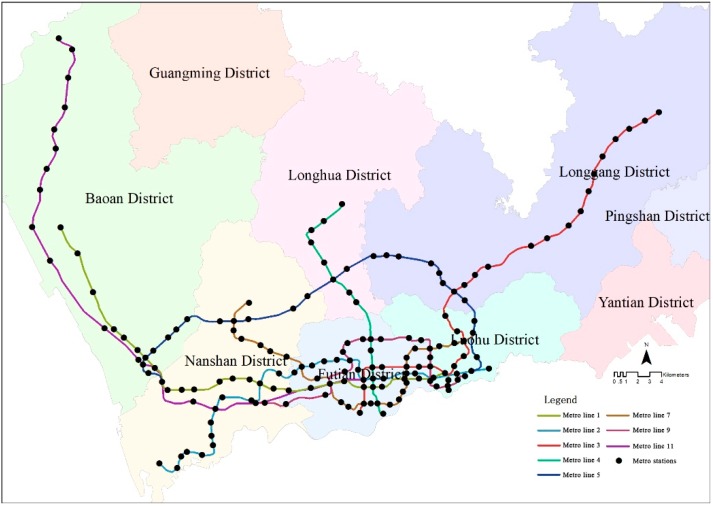
Shenzhen metro lines and stations.

Shenzhen is an emerging megacity in Guangdong Province in South China. It is also a “young” city that underwent rapid urban development from a tiny fishing village in 1979 to a massive modern city with China’s Open-Door Policy. Due to its limited land resources and growing population, the metro system and other types of public transportation have been intensively developed. By 2017, eight metro lines had been built and opened, including 167 metro stations (Figure 1). All metro stations were included in this study.

### 3.2. Data Source

#### 3.2.1. Cycling Data

Shenzhen’s first free-floating bicycle-sharing system began in October 2016. More than 890,000 bicycles have been put into service, and the number of registered users surpassed 20 million in December 2017. The purpose of about 50% of trips is to commute to or from work or school [55]. More than 65% of public bicycles are distributed around metro stations, which suggests that cycling serves as an effective feeder mode to the metro system.

We obtained cycling data for approximately 20 million trips from a large bicycle-sharing company. The data consist of all cycling trips in Shenzhen over a 14-day period from 1 to 14 December 2017, including the location (latitude, longitude) and the time stamps for the origin and destination of every trip. The weather during that period was mild (sunny and 16 °C to 25 °C, a typical temperature except summer), which allowed us to exclude the effects of weather on cycling.

Because we focused on cycling destination accessibility around metro stations, all bicycle-metro transferring trips were selected from the original data. The data processing consisted of three steps: Data cleaning, selection of bicycle-metro transferring trips, and assignment of these trips to each metro station.

Cycling trips with missing trip information or an abnormal duration (<1 min or >30 min), speed (>3 m/s), or distance (>5 km) were excluded. Because cycling trajectory and its distance were not included in the original dataset, the cycling distance was inferred by Euclidean distance between cycling starting point and ending point. If the starting point of trip n is A (*X_b_*, *Y_a_*) and the ending point is B (*X_b_*, *Y_b_*), the cycling distance *D_A,B_* for this trip is shown as Equation (2).

(2) $$D_{A,B}=\sqrt{{\left(X_{a}-X_{b}\right)}^{2}+{\left(Y_{a}-Y_{b}\right)}^{2}}$$

**Figure 2:**
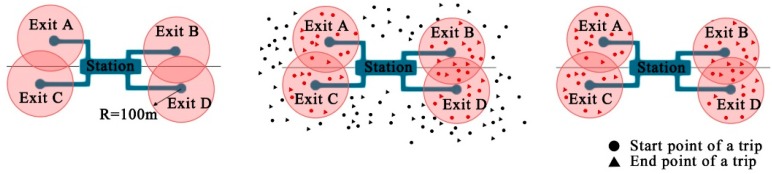
Process of identifying bicycle-metro integration trips.

The second step was the selection of bicycle-metro transferring trips (Figure 2). The cycling trips whose origin or destination lay within 100 m of any metro station entrance were considered bicycle-metro transferring trips. Finally, the selected trips were assigned to the nearest metro station, and the number of trips is calculated for each station.

#### 3.2.2. Points of Interest (POIs)

A database of POIs was obtained from Baidu Map using an API interface in December 2017 (Baidu Inc., Beijing, China). The data consisted of six categories of facilities: Residential, commercial, working, leisure, park, and public transportation POIs. The residential POIs consist of all residence communities and urban villages. The working POIs contain all companies and industry zones. The commercial POIs include shopping centers and malls. The park POIs consist of all kinds of parks and famous scenic spots. The leisure POIs refer to sports facilities, cinemas, and theaters. The public transportation POIs include bus stations. Those POIs represent the destinations or origins of cycling trips that are closely related to our daily life around metro stations.

### 3.3. Distance-Decay Function

Distance decay is a transportation planning method used to model travel behaviors in cities. Distance-decay functions are usually estimated from the distribution of sample data to represent changes in people’s willingness to cycle as the trip distance increases. Exponential functions are usually recommended for short trips as in Equation (3) [19].

(3) $$P\left(d\right)=e^{-\beta d}$$

where *P*(*d*) is the cumulative percentage of trips with a distance of at least *d* or the value of the trip distribution, and *β* is the distance-decay parameter to be estimated. The decay parameter indicates the steepness of the decline in the percentage of trips of at least a given distance. The cumulative distribution was used because people prefer shorter walking trips to longer trips. For example, if a person can walk 500 m, he or she can also walk 300 m.

However, we argue that bicycle-metro transferring trips may differ from walking behavior. The willingness to engage in metro-related cycling may increase within a certain distance threshold. In this situation, the distance-decay function cannot accurately represent the change in people’s willingness to cycle as the cycling distance increases.

**Figure 3:**
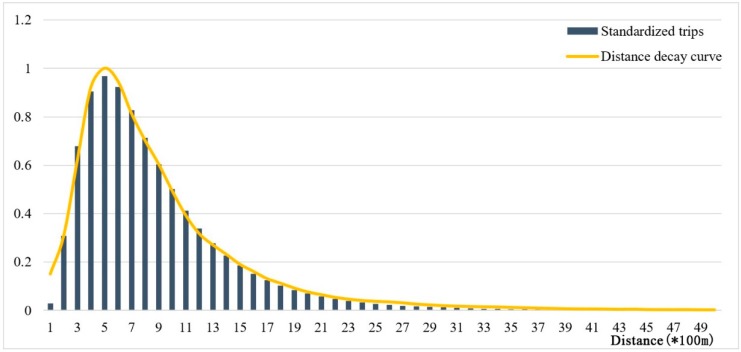
Distribution of cycling trips and fitting curve by trip distance.

To explore the new distance-decay function with the bicycle-metro trip data and test the accuracy of our findings, we randomly selected 10% of the samples from the original bicycle-metro trip data for fitting curves and used the remaining 90% of the data to check the fitting results. The distribution of the frequency of bicycle-metro trips was plotted in 100 m distance bands up to 5000 m; the distribution of standardized trips is shown in Figure 3. A lognormal distribution was found. As hypothesized, the frequency of cycling trips increased within 500 m and decreased beyond 500 m. A logarithmic normal distribution function was used to estimate the frequency of cycling trips. The distance-decay function is as shown in Equation (4).

(4) $$f\left(x\right)=\alpha \frac{1}{\sqrt{2\pi }\sigma x}e^{-\frac{{\left(\ln x-\mu \right)}^{2}}{2\sigma^{2}}}$$

where *x* is the cycling distance, and *α*, *σ*, and *μ* are the values of the parameters to be estimated, which together determine the shape of the curve.

An Excel spreadsheet with visual basic code was used to fit the curve. The dependent variable was the number of trips in each distance interval. The distance values are taken by selecting the maximum point of each distance interval.

### 3.4. Measuring Cycling Accessibility

Gravity-based measures are used in accessibility measures. We define each station’s cycling accessibility as Equation (5).

(5) $$A_{i}=\sum_{j=0}^{n}O_{i,j}f\left(d_{i,j}\right)$$

where *A_i_* represents the cycling accessibility of metro station *i*; *d_i,j_* represents the distance of *j* facilities around metro station *i*; *O_i,j_* is the facility *j* in metro station *i*; and *f*(*d_i,j_*) is the cycling distance-decay function calculated in Equation (4), which represents the attenuated values that correspond to various distances. The logic behind Equation (5) is the cumulative likelihood of cyclists reaching a variety of facilities when considering the effect of distance impedance.

The distance between a variety of facilities and metro stations was calculated using the nearest-facilities method in ArcGIS 10 (Esri Inc., Redlands, CA, USA). In addition, we excluded any facilities beyond 2.5 km from the nearest metro station because the number of bicycle-metro trips changes little beyond 2.5 km and 95% of bicycle-metro trips fall within this threshold.

### 3.5. Modeling the Number of Bicycle-Metro Trips with Proposed Cycling Accessibility Measure

**Figure 4:**
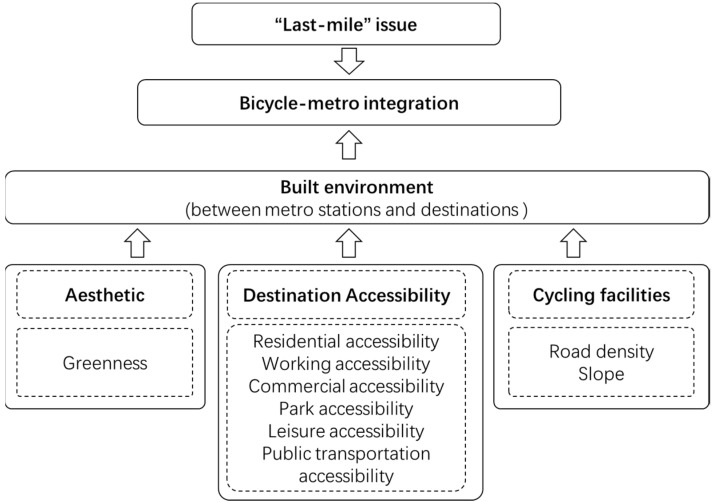
The research framework.

According to existing evidence, cycling accessibility, cycling facilities, and aesthetic factors all affect bicycle-metro integration (Figure 4) [9,23,24,25]. Cycling accessibility is believed to be the primary determinant of transit use [17]. Six different daily aspects of cycling accessibility were selected in this study: Residential accessibility, work accessibility, commercial accessibility, park accessibility, leisure accessibility, and public transportation accessibility (Table 1). All of these facilities were important destinations or origins connecting with metro stations by bicycles.

In addition, cycling facilities are directly related to cycling. A low terrain slope and high road density may have a positive effect on the cycling rate [56,57]. The road density is calculated as the length of all roads in an area. Besides, aesthetic factors are essential in encouraging cycling behavior. Urban greenness, as an aesthetics factor, may also affect the cycling rate [38]. The normalized difference vegetation index (NDVI) was used to quantify the amount of vegetation with 10-m resolution satellite imagery. It measures as Equation (6).

(6) $$NDVI=\frac{NIR-Red}{NIR+Red}$$

where NIR represents the reflection value in near-infrared band, and Red represents the reflection value of red band.

A regression model was used to model the bicycle-metro trips with all the independent variables above, including variables of the proposed cycling accessibility, cycling infrastructure and aesthetic. The remaining 90% of bicycle-metro trips which has been removed the 10% of samples of original bicycle-metro trips used for the fitting curve were as the dependent variable. The regression formula is shown by Equation (7).

(7) $$\begin{array}{l} Num=\beta_{0}+\beta_{1}\times RA+\beta_{2}\times WA+\beta_{3}\times CA+\beta_{4}\times PA+\beta_{5}\times LA+\beta_{6}\times PTA+\beta_{7}\times RD\\ +\beta_{8}\times S+\beta_{9}\times G+\varepsilon \end{array}$$

where *Num* is the number of bicycle-metro trips; *β*_0_, *β*_1_, *β*_2_, *β*_3_, *β*_4_, *β*_5_, *β*_6_, *β*_7_, *β*_8_, *β*_9_ is the parameter to be determined; and *ε* is the random variable.

To examine the performance of the proposed cycling accessibility with a lognormal distribution function, three regression models with different measurements of cycling accessibility were built. Model 1 used accessibility without a distance-decay function, which means that the willingness of cycling to facilities at different distances from metro stations had the same weight. Model 2 used accessibility with an exponential distance-decay function [44]. The willingness of cycling to different facilities decreased with the distance between facilities and metro stations. The distance-decay parameter has been estimated to be 0.00041641, according to a previous study [44]. Model 3 used accessibility with our logarithmic normal distribution function, which means that the willingness of cycling to facilities increased with distance when it was less than 500 m, and then decreased with distance when it was more than 500 m. 

In the three models, the only difference is the way of calculating cycling accessibility (Table 2), all other factors (e.g., cycling infrastructure and aesthetic) were the same across these models.

## 4. Results

### 4.1. Characteristics of Cycling Distance Decay

With the aid of our visual basic code, the parameters of logarithmic normal distribution decay function were estimated (*α* = 10.369, *σ* = 0.685, and *μ* = 2.087), and the goodness of fitness for the estimation is 0.99. The distance-decay function can be given as follows:

(8) $$f\left(d_{i,j}\right)=10.369\frac{1}{\sqrt{2\pi }0.685d_{i,j}}e^{-\frac{{\left(\ln d_{i,j}-2.087\right)}^{2}}{2{\left(0.685\right)}^{2}}}$$

where *d_i,j_* represents the distance between metro station *i* and destination *j*. The curve *f*(*d_i,j_*) represents the change in people’s willingness to cycle as the transferring distance increased. Observation of the curve reveals that 500 m was an important turning point, that is, the most popular cycling distance for transferring was 500 m. Within the range of 100 to 500 m, people’s willingness to ride gradually increased as the distance increased. Their willingness then slowly decreased as the distance increased, and it approached 0 at 5 km. The fitting curve was shown in Figure 3.

### 4.2. Mapping Cycling Destination Accessibility

**Figure 5:**
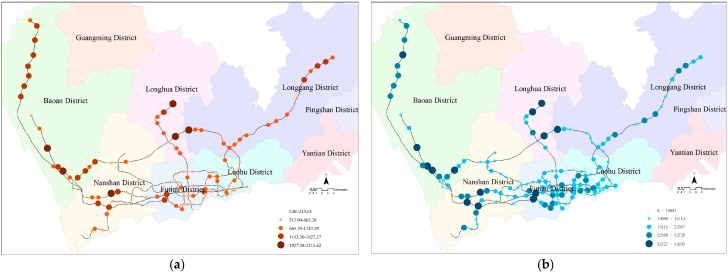
(**a**) Cycling accessibility in each metro station; (**b**) number of bicycle-metro trips in each metro station.

According to the logarithmic normal distribution decay function calculated above, the accessibility was computed for the six types of facilities. The overall accessibility of each station is the sum of the accessibility for the six types of facilities. The overall cycling accessibility of each station was visualized in ArcGIS (Figure 5a), as was the total number of bicycle-metro trips (Figure 5b).

We found that the metro stations in Nanshan, Futian, and Luohu districts had lower cycling accessibility than the stations in Baoan, Longhua, and Longgang districts. Nanshan, Futian, and Luohu districts, collectively referred to as the “Special Economic Zone” (SEZ), were developed earlier, and Baoan, Longhua, and Longgang districts, located outside the SEZ, were developed later. Hence, the metro stations and POIs in the SEZ are densely distributed, and the transferring distances are relatively short. In this situation, people prefer to walk to connecting metro stations. In contrast, metro stations and POIs outside the SEZ are sparse, and the transferring distances are relatively long. In this situation, people prefer cycling as a transfer mode. In addition, some interchange metro stations, such as Chegongmiao, Huaqiangbei, and Houhai stations, had greater accessibility. However, interchange stations that link to high-speed rail stations, such as Shenzhenbei and Luohu, had lower accessibility. The interchange stations have more passengers and more kinds of facilities than normal stations. Hence, people can reach more facilities by cycling, and the bicycle-metro trips are longer, because the passengers at high-speed rail stations mainly transfer within the stations or integrate with taxis and buses. Thus, a variety of transfer options may affect the choice of cycling.

### 4.3. Regression Models

The regression results are shown in Table 3. The model with a logarithmic normal distribution decay function (i.e., Model 3) had a greater association with the number of bicycle-metro trips (R^2^ = 0.445) than the model without distance decay (R^2^ = 0.365) or the model with exponent decay (R^2^ = 0.411). The results indicate that Model 3 can explain 44.5% of the variance in the number of bicycle-metro trips. 

In addition, the working accessibility and leisure accessibility were both significant at <0.01 level in the three models, which means that working accessibility and leisure accessibility were both crucial factors influencing bicycle-metro integration. Road network density was also significant in all the models. 

## 5. Discussion

Cycling is a viable transfer mode to promote the use of public transit, and a policy of bicycle-transit integration has been actively advocated by many governments in both developed and developing countries. Destination accessibility is believed to be a critical factor that can facilitate or hinder transit-transferring cycling behaviors. However, destination accessibility was not rigorously defined in previous studies because walking and cycling behaviors were not distinguished. In this study, we found a lognormal distribution decay curve for bicycle-metro trips of a large public bicycle-sharing program in Shenzhen, China. The destination accessibility with a lognormal distribution distance-decay function was proposed by considering the competition between cycling and walking within 500 m around each metro station. Our new accessibility measure had greater associations with the number of bicycle-metro trips than existing models. More specifically, this study has three major findings.

First, the superior performance of our new accessibility measure indicates that the distance decay of bicycle-metro trips conforms to a lognormal distribution. The findings of this study differ from those of previous studies that the distance decay of both cycling and walking trips fits an exponential curve [21,44,58,59]. The difference may be explained by the data sources for the cycling trips. Previous studies often collected cycling data with a survey or travel diary, which are subject to limitations in sample size and site coverage. In contrast to previous studies, big data from more than three million cycling trips from a dockless bicycle-sharing program were used in this study. The large sample size gives us adequate detection power to delineate the distance-decay functions of bicycle-metro trips.

In addition, walking and cycling may compete as transfer modes to transit service within 500 m. From the cycling distance-decay curve (Figure 3), we found an upward trend in the number of cycling trips between 100 and 500 m, which indicates that the willingness to choose cycling as a transfer mode to transit increases within this distance threshold. In contrast, previous studies suggested that the willingness to walk always decreases as the distance increases. Hence, we infer that the relationship between the choice of cycling or walking is competitive within 500 m of a metro station. This competition can be explained by economic cost, time cost, and other factors regarding the use of public bicycles. The bicycle-sharing program charges rent, and it may take time to locate a bicycle. Therefore, for short trips within 500 m around a metro station, people tend to prefer walking to cycling as a transfer mode. Thus, in the area within 500 m of a metro station, we recommend that greater attention should be paid to the creation of a pedestrian-friendly environment.

Second, the working POI accessibility, leisure POI accessibility, and road density are important built environment indicators that affect bicycle-metro trips. Similar results were found in that the availability of cycling infrastructure [31,60] and accessibility to jobs [61] can promote the use of cycling. However, residential accessibility was not associated with the number of cycling trips in this study, which is in contrast to previous findings [53]. This contrast may be due to the limitations of our residential POI data. The residential POIs were represented by points regardless of the number of residents or the size of the housing estate. For example, in Shenzhen, nearly 11 million people, approximately half of the total population, live in 320 urban villages [62]. The building density in these urban villages is higher and the living environment is poorer than in normal residence communities, and many low-income groups live there. However, these urban villages were also represented by a single POI, although some villages, such as Baishizhou, Xiasha, and Gangxia, accommodate more than 100,000 people. In this situation, the number of residents was misrepresented. Future studies should consider the number of residents.

Third, the threshold of bicycle-metro trips was higher than that in previous studies. Most studies used the 85th-percentile access distance to confirm the catchment area. In this paper, the 85th-percentile distance of all bicycle-metro trips was about 1.5 km, which is not consistent with previous conclusions generated by survey data. For example, the access distances to metro stations for cyclers in Beijing fall mainly between 0.4 and 1.4 km, which is less than our finding [63].

However, the access distance above is commonly obtained from a self-reported distance or a distance calculated from an algorithm-identified route with a reported origin/destination and stop location, which may lead to an inaccurate result. This problem was improved in this study using the bicycle-sharing big data. We defined the threshold of bicycle-metro trips in Shenzhen as about 2.5 km, which covers 95% of cycling trips, because the number of bicycle-metro trips changed little beyond 2.5 km and 95% of bicycle-metro trips are within this threshold. The findings from this study have important policy and planning implications; by providing a safe and comfortable cycling environment and well-established public bicycle-sharing systems, we can promote bicycle–transit integration and thus improve both cycling and transit usage in the 2.5-km transit-catchment area. Further validation about the 2.5 km threshold is needed to confirm the generalizability of this finding.

This study has both strengths and limitations. We distinguished cycling and walking as transfer modes to and from metro stations. The competition between cycling and walking within 500 m of metro stations help us to develop a new cycling destination accessibility model with a lognormal distribution decay curve. Furthermore, we used the big data from more than three million trips of a public dockless bicycle-sharing program, which addressed many limitations of previous cycling data collected from surveys or travel diaries, such as limited sample size and study area. The limitations of this study arise mainly from the nature of the bicycle-sharing data and the lack of metro ridership data. First, we cannot collect individual factors (e.g., age, gender, income), which prevent us from controlling for the potential influence of those factors on cycling behavior. Second, the decision to cycle is affected by the availability of public bicycles around metro stations. The tidal phenomenon of sharing bicycles (e.g., a person may not find a bicycle during peak hours) may affect the number of bicycle-metro trips. Third, the dataset does not include summer season data, hence we cannot understand the effect of weather on cycling behavior. Forth, the metro ridership data is also an important factor affecting the cycling transferring trips, which may lead a relatively weak explanation in the regression model. In addition, we assume that the six types of facilities have the same distance-decay function and weight in the accessibility model process. Although the geographic locations of the trip destination were collected, the destinations of our data were uncertain (e.g., which POI was visited when multiple POIs were located around the geographic locations of trip destinations). Future studies may combine various data sources, such as travel diary and geocode public cycling-sharing data to address these issues.

## 6. Conclusions

In this study, we measured cycling destination accessibility with the big data from more than three million trips on a public bicycle-sharing system. We found that the distance-decay function associated with cycle-metro trips conforms to a lognormal distribution. Most specifically, the frequency of cycle-metro trips increases with distance within 500 m of a metro station and then decreases beyond 500 m. The pattern differs significantly from that of walking behavior. Our results also show that our proposed destination accessibility model outperforms existing models in fitting the frequency of cycle-metro trips. Furthermore, the optimal distance of bicycle-metro trips in Shenzhen is about 2.5 km, or around 15 min by bicycle. Hence, promotion of a policy of cycle-metro integration can effectively extend metro service coverage areas 800 to 1500 m from metro stations. By proposing robust and validated metrics for cycling destination accessibility, the findings of this study may help policymakers and planners create a cycling-friendly built environment and thus lead to better cycle–metro integration.

## Figures and Tables

**Table 1:** Independent Variables.

Dimensions of Built Environment	Indicators	Variable	Measurement
Cycling accessibility	Residence accessibility	RA	The sum of the distances from all residence points of interest (POI) within a 2.5 km buffer to the metro station considering distance decay, as Equation (5)
	Work accessibility	WA	The sum of the distances from all work POI within a 2.5 km buffer to the metro station considering distance decay, as Equation (5)
	Commercial accessibility	CA	The sum of the distances from all commercial POI within a 2.5 km buffer to the metro station considering distance decay, as Equation (5)
	Park accessibility	PA	The sum of the distances from all park POI within a 2.5 km buffer to the metro station considering distance decay, as Equation (5)
	Leisure accessibility	LA	The sum of the distances from all leisure POI within a 2.5 km buffer to the metro station considering distance decay, as Equation (5)
	Public transportation accessibility	PTA	The sum of the distances from all public transportation POI within a 2.5 km buffer to the metro station considering distance decay, as Equation (5)
Cycling infrastructure	Road density	RD	Length of all roads divided by buffer area with a 2.5 km radius
	Slope	S	The average slope in the 2.5 km buffer
Aesthetic	Greenness	G	The average NDVI value in the 2.5 km buffer

**Table 2:** Calculation of Cycling Accessibility in Three Models.

Model	Calculation of Cycling Accessibility
Model 1	$A_{i}=O_{i,j}$
Model 2	$A_{i}=O_{i,j}\times d_{i,j}\times e^{0.00041641}$
Model 3	$A_{i}=O_{i,j}\times \alpha \frac{1}{\sqrt{2\pi }\sigma x}e^{-\frac{{\left(\ln x-\mu \right)}^{2}}{2\sigma^{2}}}$

**Table 3:** Results of Regression Models.

Dimension of Built Environme	Indicator	Model 1			Model 2			Model 3		
		B	S.E.	Sig	B	S.E.	Sig	B	S.E.	Sig
Destination accessibility	Resident	−4.80	14.47	0.74	−11.27	18.66	0.55	−25.90	18.71	0.17
	Working	4.44	1.39	<0.01	6.48	2.01	0.00	7.88	2.34	<0.01
	Commercial	−47.25	55.93	0.40	−5.23	81.70	0.95	102.65	94.06	0.28
	Park	−110.31	308.58	0.72	−191.05	390.27	0.63	−328.41	402.30	0.42
	Leisure	274.54	65.29	<0.01	366.97	85.36	0.00	396.27	94.02	<0.01
	Public transport	−0.54	9.02	0.95	6.03	12.30	0.63	12.89	13.90	0.36
Cycling infrastructure	Road density	39.75	18.03	0.03	37.54	17.24	0.03	43.17	16.49	0.01
	Slope	−537.22	613.71	0.38	−576.88	589.06	0.33	−539.99	571.07	0.35
Aesthetic	Greenness	12,375.89	30,664.02	0.69	13,895.52	29,635.42	0.64	12,119.36	28,622.08	0.67
Model fit information	Adjusted R^2^	0.365			0.411			0.445		
	Error of std. estimate	9198.33			8857.83			8593.16		
	Significance	*p* < 0.01			*p* < 0.01			*p* < 0.01		

* B: Beta; * S.E.: Standard Error; * Sig: Significance.
